# Establishment of interleukin‐18 time‐resolved fluorescence immunoassay and its preliminary application in liver disease

**DOI:** 10.1002/jcla.23758

**Published:** 2021-03-15

**Authors:** Li Zhang, Xiumei Zhou, Yaping Dai, Chunyan Lv, Jian Wu, Qingqing Wu, Ting Li, Yigang Wang, Penguo Xia, Hao Pei, Biao Huang

**Affiliations:** ^1^ College of Life Sciences and Medicine Zhejiang Sci‐Tech University Hangzhou China; ^2^ Wuxi No. 5 People’s Hospital Wuxi China; ^3^ Department of Laboratory Medicine The First People’s Hospital of Yancheng City Yancheng China

**Keywords:** ELISA, interferon‐γ, interleukin 18, liver disease, time‐resolved fluorescence immunoassay

## Abstract

**Background:**

To establish a time‐resolved fluorescence immunoassay of interleukin (IL)‐18 (IL‐18‐TRFIA) and detect its concentration in different liver disease serum samples.

**Methods:**

The IL‐18 coating antibody and the Eu^3+^‐labeled detection antibody were used for the IL‐18‐TRFIA to detect serum IL‐18 concentration in patients with liver cancer, hepatitis B, hepatitis C, autoimmune hepatitis, fatty liver disease, and healthy controls. The double‐antibody sandwich method was used and methodological evaluation was performed.

**Results:**

The average intra‐ and inter‐assay coefficient of variation for IL‐18‐TRFIA was 4.80% and 5.90%, respectively. The average recovery rate was 106.19 ± 3.44%. The sensitivity (10.96 pg/mL) was higher than that obtained using the ELISA method (62.5 pg/mL). The detection range was 10.96–1000 pg/mL. IL‐6 and galectin‐3 did not cross‐react with IL‐18‐TRFIA. The serum concentration of IL‐18 was (776.99; 653.48–952.39 pg/mL) in hepatitis C, (911; 775.55–1130.03 pg/mL) in fatty liver, (1048.88; 730.04–1185.10 pg/mL) in liver cancer, and (949.12; 723.70–1160.28 pg/mL) in hepatitis B. Moreover, IL‐18 serum levels were significantly higher in patients than the healthy controls (483.09; 402.52–599.70/mL) (*p* < 0.0001). Autoimmune hepatitis with a serum IL‐18 concentration of 571.62; 502.47–730.31 pg/mL was not significantly different from the healthy controls (*p* > 0.05).

**Conclusion:**

We established a highly sensitive IL‐18‐TRFIA method that successfully detected serum IL‐18 concentrations in different liver diseases. Furthermore, IL‐18 serum concentration was higher in patients with liver cancer, hepatitis C, hepatitis B, and fatty liver disease compared to healthy controls.

## Graphical abstract

Comparison of the serum IL‐18 concentrations between five groups of liver disease patients and healthy controls. The average concentration of IL‐18 in the serum of patients with liver cancer was the highest.

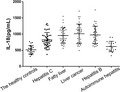

## 1 INTRODUCTION

Interleukin 18 (IL‐18), also designated interferon‐γ (IFN‐γ) inducing factor, is a single nonglycosylated peptide chain with a molecular weight of 18 kDa that was first identified as a cytokine in 1996.^1^ IL‐18 has been reported in most cell types, including endothelial cells, macrophages, dendritic cells, vascular smooth muscle cells, and Kupffer cells.^2^, ^3^

The cytoplasmic IL‐18 precursor is an inactive 192 amino acid polypeptide with a molecular weight of 24 kDa, which becomes cleaved by group I or inflammatory caspases.^4^ The active 18 kDa mature IL‐18 then exerts proinflammatory and anti‐inflammatory effects.^5^ A comprehensive description of its roles in numerous diseases such as nephropathy,^6^ leukemia,^7^ atherosclerosis,^8^ obesity,^9^ diabetes,^10^ and HIV^11^ has been previously reported. According to the literature, the expression of IL‐18 is elevated in the serum of patients with hepatitis B^12^ and liver cancer^13^ compared to healthy controls. Therefore, we aimed to establish a highly sensitive time‐resolved fluorescence immunoassay (TRFIA)^14^ and evaluate its application for detecting IL‐18 in the serum of liver cancer, hepatitis B, hepatitis C, autoimmune hepatitis, and fatty liver disease patients.

## 2 MATERIALS AND METHODS

### 2.1 Reagents and instruments

Coating antibody M02 and detection antibody M01 were purchased from Chilue Biotechnology Co., Ltd.. These two kinds of monoclonal antibodies were specific for IL‐18 epitopes. IL‐18 antigen standard and ELISA kit (Yiqiao Shenzhou Biotechnology Co., Ltd.), bovine serum albumin (BSA, Saiguo Biotechnology Co., Ltd.), europium isothiocyanate benzyl diethylenetriaminetetraacetic acid (DTTA‐Eu, Boshi Biotech Co.), and T cell immunoglobulin protein Mucin‐3 (TIM‐3) detection kit were provided by our laboratory. Other necessary reagents and instruments used in this study were as follows: the SepHadex‐G50 column (Xibao Biotechnology Co., Ltd.), Ultracel‐50 K ultrafiltration tube (Millipore), 96‐well enzyme label plate (Yunpeng Technology Development Co., Ltd.), time‐resolved fluorescence immunoassay analyzer (DR6608, Foshan Daan Medical Equipment Co., Ltd.), plate washer (DEM‐3, Darui Biotechnology Co., Ltd.), electric incubator (DNP‐9022, Jinghong Experimental Equipment Co., Ltd.), automatic fraction collector (BSZ‐160, Qingpu Huxi Instrument Factory), and micro oscillator (Kangjian Medical Products Co., Ltd.).

### 2.2 Serum samples

Blood samples were collected from healthy individuals and five different types of liver disease (liver cancer, hepatitis B, hepatitis C, autoimmune hepatitis, and fatty liver disease) from the Fifth People's Hospital of Wuxi (*n* = 186 samples). The samples were centrifuged at 1200 × *g* for 5 min to obtain the serum, which was then stored at −20°C. No hemolysis or lipid turbidity steps was included in the preparation of serum samples. The selection criteria for healthy subjects included: negative results for HBs antigens and hepatitis C virus (HCV) antibodies, and normal liver function. The research project was approved by the Ethics Committee of Wuxi Fifth People's Hospital (ethical code: 2020–023–1).

### 2.3 Buffer preparation

Coating buffer (50 mmol/L Na_2_CO_3_‐NaHCO_3_, pH 9.6), labeling buffer (50 mmol/L Na_2_CO_3_‐NaHCO_3_, pH 9.0), elution buffer (50 mmol/L Tris‐HCl, containing 0.9% NaCl, 0.05% proclin‐300, and 0.2% BSA, pH 7.8), blocking buffer (50 mmol/L Tris‐HCl, containing 0.9% NaCl, 1% BSA, and 0.05% NaN_3_, pH 7.8), enhancement solution (15 μmol β‐NTA, 50 μmol trioctylphosphine oxide, 1 mL Triton X‐100/L, pH 3.2), phosphate‐buffered saline (PBS) solution (0.01 mol/L sodium phosphate buffer, containing 0.9% NaCl, pH 7.4), antigen standard dilution (30% FBS, 0.1% BSA, and 0.05% Tween20 dissolved in PBS, pH 7.4), analysis buffer (50 mmol/L Tris‐HCl, containing 0.9% NaCl, 0.2% BSA, 0.01% Tween‐20, 20 μM DTPA, and 0.05% NaN_3_, pH 7.8), and washing solution (50 mmol/L Tris‐HCl, containing 0.9% NaCl, 0.02% Tween20, and 0.01% Proclin 300, pH 7.8) were prepared.

### 2.4 Preparation of solid‐phase coated antibodies

The coating antibody was diluted to 4 μg/mL with 50 mmol/L of carbonate buffer (pH 9.6), after which 100 μL of solution was added to each well of a 96‐well microtiter plate and incubated overnight at 4℃. Next, we removed the coating antibody solution and washed the plates with DEM‐3 plate washer. Next, we added 150 μL of sealing solution to each well, sealed the plates, and incubated them at 25℃ for 2 h. Lastly, the sealing solution was removed, the plate was washed, vacuum dried, vacuum packed in an aluminum foil bag, and stored at −20℃.

### 2.5 Preparation and purification of Eu^3+^ labeled antibody

A total of 0.3 mg of labeled antibody was added to the Millipore centrifuge tube with a filter membrane and centrifuged at 2862 × *g* for 8 min. Following centrifugation, the pellet was washed eight times with 300 μL of labeling buffer. Next, 50 μL of labeled antibody and 100 μg of europium labeling reagent were thoroughly mixed and incubated at 28°C overnight with constant shaking. We then used the SepHadex‐G50 chromatography column to separate, purify, and elute the eluent, while simultaneously collecting the effluent (2 mL/tube). Next, 5 μL of stock solution and 100 μL of enhancement solution were added to each tube to measure the fluorescence coefficient (counts per second, CPS). After combining the first peak tube, it was stored in the freezer at −20°C.

### 2.6 TRFIA evaluation of IL‐18

TRFIA detection of IL‐18 was carried out using the two‐step double‐antibody sandwich method. First, we added a volume of 100 μL IL‐18 antigen standard (62 pg/mL, 125 pg/mL, 250 pg/mL, 500 pg/mL, and 1000 pg/mL) or serum to each antibody‐coated well. After a 1 h incubation period at 37℃, the plate was washed twice using a plate washer and patted dry. Next, we added 100 μL of diluted Eu^3+^ detection antibody (1:400) to each well and incubated the plate at 37°C for 1 h. The plate was washed 6 times using the plate washer and patted dry. Next, we added 100 μL of enhancement solution to each well, and agitated the plate on a micro shaker for 5 min. The fluorescence coefficient (CPS) was subsequently measured. The method used for determining the concentration of IL‐18 in serum samples was the same as that described for antigen standards.

#### 2.6.1 Accuracy

We selected three IL‐18 antigen standards with low (125 pg/mL), medium (250 pg/mL), and high concentrations (500 pg/mL), and used the established IL‐18‐TRFIA method for intra‐assay and inter‐assay precision testing.

#### 2.6.2 Sensitivity and specificity

To identify the lowest concentration of the analyte to be reliably detected from the background noise, we took the mean and standard deviation (SD) of the values at zero‐concentration points of the ten standard curves, obtaining the mean +2 × SD.^15^ The sensitivity of the method was then calculated based on the standard curve. IL‐6 and galectin‐3 were used as interfering substances to measure the cross‐reaction rate.

#### 2.6.3 Recovery rate

To evaluate the serum recovery rate, two serum samples with known concentrations (176.64 pg/mL and 431.34 pg/mL) were mixed with an IL‐18 standard (1000 pg/mL) at a ratio of 9:1. After adding the IL‐18 standard, theoretically, the measured concentration values for the two samples should be 258.97 pg/mL and 488.21 pg/mL, respectively. The recovery rate (%) of IL‐18 was calculated as follows: actual concentration/theoretical concentration ×100.

#### 2.6.4 Comparison of TRFIA method and ELISA method

A total of 36 serum samples containing different concentrations of IL‐18 were selected. First, the IL‐18 serum concentration was determined using ELISA according to the manufacturer's instructions. The results were then compared to those obtained using the TRFIA method.

#### 2.6.5 Clinical application of the TRFIA method

We next aimed to evaluate the preliminary clinical application of the TRFIA method. To this end, we used serum samples from patients diagnosed with hepatitis C (*n* = 42), fatty liver disease (*n* = 36), liver cancer (*n* = 36), autoimmune hepatitis (*n* = 17), and hepatitis B (*n* = 35) provided by Wuxi Fifth People's Hospital, and healthy controls serum samples (*n* = 20). The TRFIA method measured IL‐18 concentration in the serum, as well as the concentration of TIM‐3,^16^ while other biochemical and clinical indicators were provided by Wuxi Fifth People's Hospital.

#### 2.6.6 Statistical analysis

The results were expressed as mean ± SD. The liver disease and healthy controls’ IL‐18 concentrations in the serum were expressed as median, 25–75 quartiles. The correlation between IL‐18 and other parameters of liver disease was analyzed using IBM SPSS Statistics 26 (IBM) software, and the graph was plotted using GraphPad Prism 6 (GraphPad software). We used Spearman's correlation to assess whether the IL‐18 concentration was correlated with clinical indicators and Jonckheere‐Terpstra test to compare the concentration of IL‐18 in the serum of patients with different liver diseases. *p*‐values < 0.05 were considered statistically significant.

## 3 RESULTS

### 3.1 IL‐18 standard curve

Following the measurement of the IL‐18 standards using the TRFIA method, we generated a standard curve fitted with a degree function. The IL‐18 standard curve equation was as follows: *y* = 28.87*x* ‐ 471.50, *p* < 0.0001, and the multiple correlation coefficient (*R* ^2^) was 0.9986.

### 3.2 Accuracy

We selected three IL‐18 antigen standards with low, medium, and high concentration levels, and used the IL‐18‐TRFIA method to test each one ten times. The average intra‐assay CV was 4.80% while the average CV between groups was 5.90%.

### 3.3 Sensitivity, detection range, and specificity

The fluorescence value of mean +2 × SD at the zero‐concentration point corresponded to a concentration of 10.96 pg/mL; thus, the detection range was between 10.96–1000 pg/mL. The measured values of IL‐6 and galectin‐3 were 0.15% and 0.48% of the theoretical values, respectively, indicating that there was no cross‐reaction with either of these proteins.

### 3.4 Recovery rate

High IL‐18 concentration standard was added to a low IL‐18 concentration serum. The average recovery rate of IL‐18 was 106.19 ± 3.44%, which was controlled between 85% and 115%.

### 3.5 Correlation between TRFIA and ELISA

**FIGURE 1:**
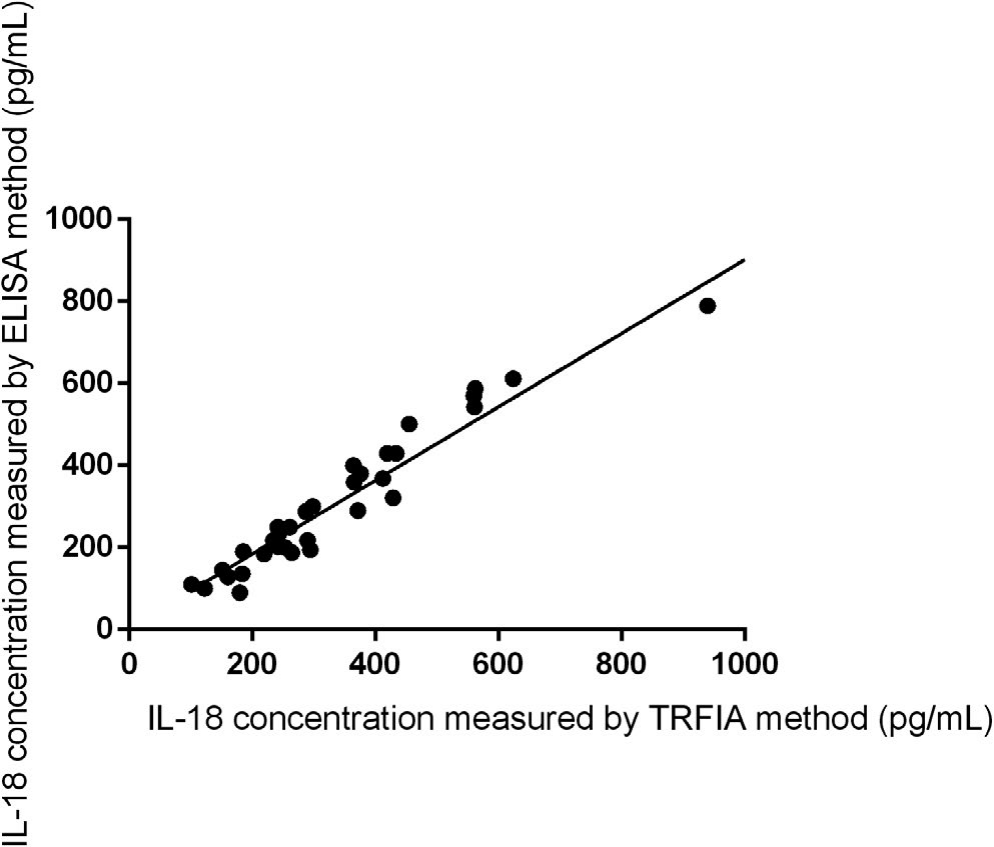
Correlation between IL‐18 concentration in serum of patients with liver disease and healthy controls measured using the TRFIA and ELISA methods (*y* = 0.8972*x* + 4.61, *R* ^2^ was 0.9596, *p* < 0.0001)

There was strong correlation between the results obtained following TRFIA and ELISA as shown in Figure 1.

### 3.6 Clinical application

**FIGURE 2:**
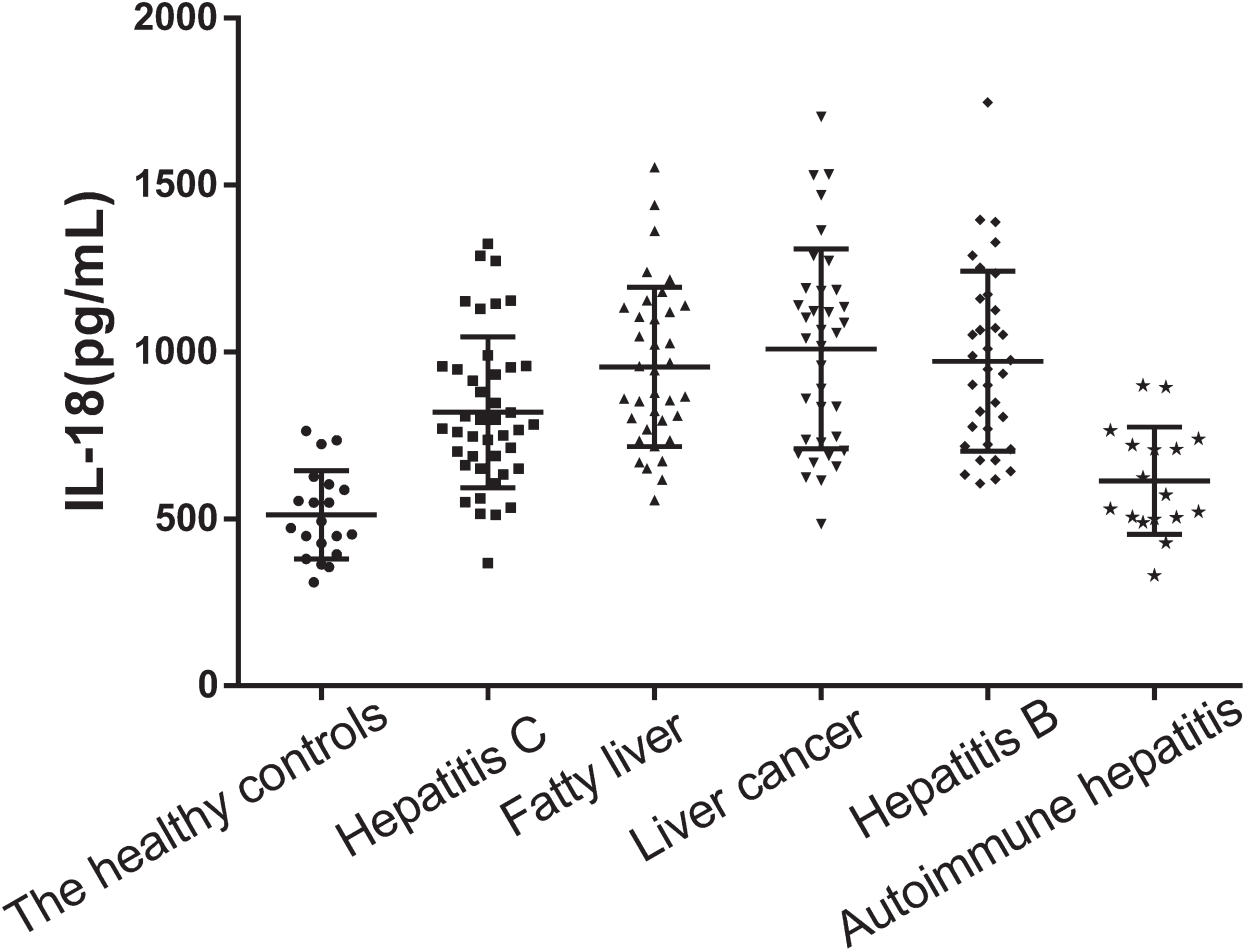
Comparison of the serum IL‐18 concentrations between five groups of liver disease patients and healthy controls

**FIGURE 3:**
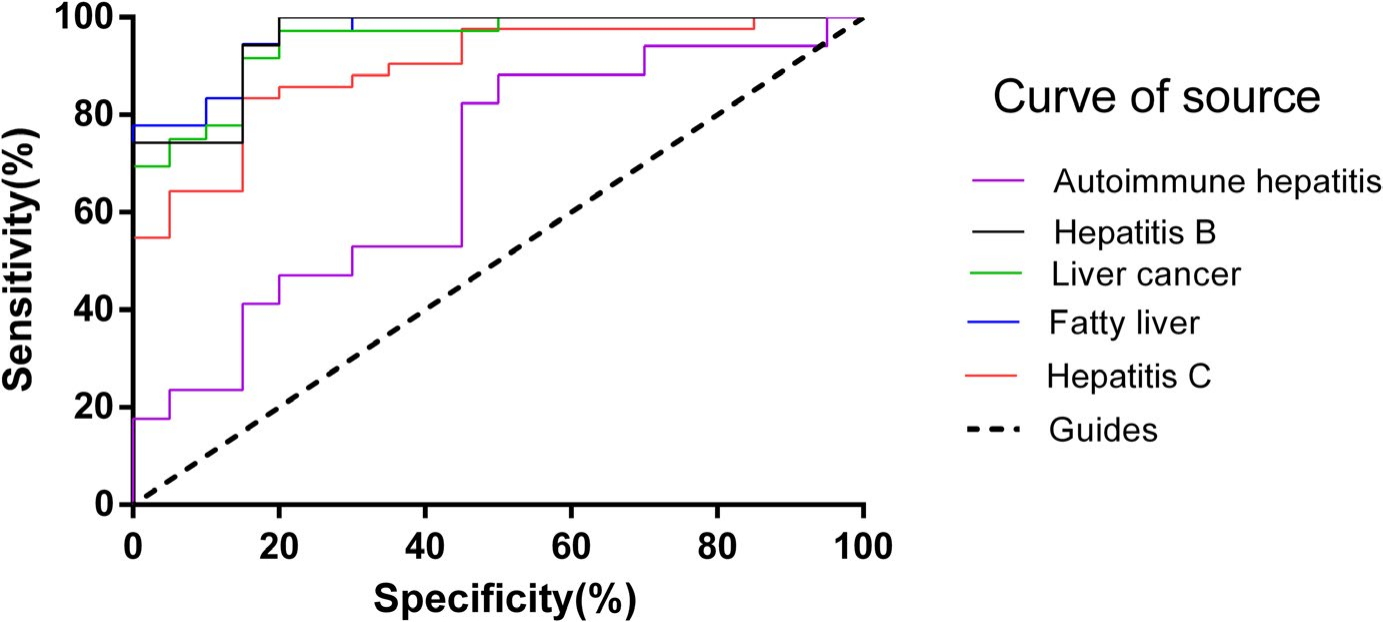
ROC curve of liver cancer, hepatitis C, hepatitis B, autoimmune hepatitis, fatty liver disease patients, and the healthy controls. (Liver cancer: AUC = 0.94, sensitivity = 97.22%, specificity = 80%, cutoff = 609.8 pg/mL, *p* < 0.0001. Fatty liver: AUC = 0.96, sensitivity = 94.44%, specificity = 85%, cutoff = 639.2 pg/mL, *p* < 0.0001. Hepatitis C: AUC = 0.89, sensitivity = 83.33%, specificity = 85%, cutoff = 629.7 pg/mL, *p* < 0.0001. Hepatitis B: AUC = 0.95, sensitivity = 94.29%, specificity = 85%, cutoff = 629.8 pg/mL, *p* < 0.0001. Autoimmune hepatitis: AUC = 0.68, sensitivity = 47.06%, specificity = 80%, cutoff = 614.1 pg/mL, *p* > 0.05)

The IL‐18 serum concentrations of liver cancer, hepatitis C, hepatitis B, and fatty liver disease patients were significantly higher than that of healthy controls. The positive rate was calculated using the IL‐18 data obtained from the 20 healthy control serum samples and a value of 775.66 pg/mL was defined as the cutoff (mean +2 × SD). The average concentration of IL‐18 in the serum of patients with liver cancer was the highest (Figure 2). The IL‐18 concentration in the serum of patients with liver cancer, hepatitis C, hepatitis B, fatty liver disease, and autoimmune hepatitis was plotted as a ROC curve (Figure 3).

The correlation between IL‐18 levels in patients with liver disease and healthy controls and routine clinical indicators provided by Wuxi Fifth People's Hospital are shown in Table 1. We found that there were significant correlations between IL‐18 and TIM‐3 (*p* < 0.01), alanine aminotransferase (ALT, *p* < 0.01), lactate dehydrogenase (LD. *p* < 0.01), direct bilirubin (DBIL, *p* < 0.05), total protein (TP, *p* < 0.05), aspartate aminotransferase (AST, *p* < 0.05), and glutamyl transpeptidase (GT, *p* < 0.05), while no correlation was observed between IL‐18 and other clinical indicators.

**TABLE 1:** Correlation between serum IL‐18 concentration in patients with liver disease and healthy controls and the clinical indicators provided by Wuxi Fifth People's Hospital

Parameters	Correlation coefficient (*r*)	*p*‐value
TIM−3 (ng/mL)	0.275	0.003**
TBIL (μmol/L)	0.169	0.174
DBIL (μmol/L)	0.251	0.042*
TP (g/L)	−0.313	0.011*
ALB (g/L)	−0.187	0.133
IG (g/L)	−0.013	0.920
ALT (U/L)	0.327	0.007**
AST (U/L)	0.298	0.015*
AST/ALT	0.048	0.701
ALP (U/L)	0.211	0.090
GT (U/L)	0.244	0.048*
LD (U/L)	0.356	0.003**
UREA (mmol/L)	0.077	0.553
CRE (μmol/L)	0.225	0.081
URIC (μmol/L)	0.143	0.275

Abbreviations: ALB, albumin; ALP, alkaline phosphatase; ALT, alanine aminotransferase; AST, aspartate aminotransferase; AST/ALT, aspartate aminotransferase/alanine aminotransferase; CRE, creatinine; DBIL, direct bilirubin; GT, glutamyl transpeptidase; IG, globulin; LD, lactate dehydrogenase; TBIL, total bilirubin; TP, total protein; UREA, urea; URIC, uric acid.

* *p* < 0.05,

** *p* < 0.01.

Since the serum IL‐18 concentration in patients with fatty liver disease was high, correlations between IL‐18 serum concentration in these patients and high‐density lipoprotein (HDL), low‐density lipoprotein (LDL), triglycerides (TRIG), and total cholesterol (TC) were analyzed (Table 2). We discovered that the IL‐18 serum concentration was positively correlated with GT level (*p* < 0.05; *r* = 0.532) and inversely correlated with HDL levels (*p* < 0.01; *r* = −0.697).

**TABLE 2:** Correlations between serum IL‐18 concentration in patients with fatty liver disease and clinical parameters provided by Wuxi Fifth People's Hospital

Parameters	Correlation coefficient (*r*)	*p*‐value
GT (U/L)	0.532	0.0490*
HDL (mmol/L)	−0.697	0.006**
LDL (mmol/L)	−0.002	0.994
TRIG (mmol/L)	0.169	0.563
TC (mmol/L)	−0.191	0.513

Abbreviations: GT, glutamyl transpeptidase; HDL, high‐density lipoprotein; LDL, low‐density lipoprotein; TC, total cholesterol; TRIG, triglyceride.

* *p* < 0.05,

** *p* < 0.01.

In patients with liver cancer, the correlations between serum IL‐18 concentration and alanine aminotransferase, GT, LD, prealbumin, a‐fucosidase, alpha‐fetoprotein, hyaluronic acid, laminin, N‐terminal type III procollagen, and type IV collagen were analyzed (Table 3). While alanine aminotransferase, GT, LD, and a‐fucosidase were significantly positively correlated with IL‐18 concentration (*p* < 0.05), prealbumin was inversely correlated with IL‐18 (*p* < 0.05). Meanwhile, the N‐terminal type III procollagen levels were most strongly correlated with the IL‐18 concentration (*p* < 0.01; *r* = 0.745).

**TABLE 3:** Correlation between serum IL‐18 concentration in patients with liver cancer and clinical parameters provided by Wuxi Fifth People's Hospital

Parameters	Correlation coefficient (*r*)	*p*‐value
ALT (U/L)	0.534	0.033*
GT (U/L)	0.562	0.024*
LD (U/L)	0.541	0.030*
PA (mg/L)	−0.614	0.015*
AFU (U/L)	0.629	0.012*
AFP (ng/mL)	0.462	0.072
HA (ng/mL)	0.300	0.370
LN (ng/mL)	0.564	0.071
P Ⅲ N P (ng/mL)	0.745	0.008**
C IV (ng/mL)	0.700	0.016

Abbreviations: AFP, Alpha‐fetoprotein; AFU, a‐fucosidase; ALT, alanine aminotransferase; C Ⅳ, type IV collagen; GT, glutamyl transpeptidase; HA, hyaluronic acid; LD, lactate dehydrogenase; LN, laminin; P Ⅲ N P, N‐terminal type III procollagen; PA, prealbumin.

* *p* < 0.05,

** *p* < 0.01.

## 4 DISCUSSION

The expression of cytokines is almost undetectable in a healthy liver; however, certain factors make the liver susceptible to cytokine‐mediated damage, such as chronic alcohol intake and obesity.^17^ Inflammatory cytokines play a classical role in the regulation of immune responses. Indeed, increasing evidence indicates that pathological changes in the liver, as well as its regeneration after injury, are mediated by cytokines, such as IFN‐γ, which have been shown to play an important role in liver damage.^18^ Moreover, the two types of T effector cells, Th1 and Th2, secrete different cytokines in humans and mice. Specifically, Th1 cells secrete primarily IFN‐γ and IL‐2 and are induced by IL‐18, while Th2 cells secrete primarily IL‐4, IL‐5, and IL‐13. The former are major players in cellular immunity, while the latter regulate humoral immune response.^19^

IL‐18 is involved in tumor formation, growth, and metastasis; while in mouse models, it has been shown to be a key role in the development of liver injury. Specifically, IL‐18 reportedly induces liver injury in a mouse model by upregulating IFN‐γ production. Endogenous IFN‐γ can enhance the secretion of IL‐18 and IL‐12, leading to aggravation of liver damage. IL‐18 also enhances the perforin‐dependent cytotoxicity of liver natural killer cells through the production of IFN‐γ, and the cytotoxicity of Th1 cells by mediating the Fas ligand.^20^

In the process of hepatitis B virus (HBV) infecting the host, human leukocyte antigen (HLA)‐DR and IL‐18 genes participate in the immune response. After HBx protein induces IL‐18 expression in the liver, it increases the release of IFN‐γ from peripheral blood monocytes in patients with chronic hepatitis B.^19^ HCV infection can cause continuous damage to the liver, and it is the main cause of liver cancer, liver cirrhosis, and liver transplantation indications.^21^ The data of Arpita et al^22^ confirmed the proinflammatory effect of IL‐18 in HCV infection, and found that the level of IL‐18 reflects the inflammation and liver damage caused by HCV. In this study, the serum levels of IL‐18 in patients with hepatitis B (949.12; 723.70–1160.28 pg/mL) and hepatitis C (776.99; 653.48–952.39 pg/mL) were both at a high level when compared to healthy controls, suggesting that high concentrations of IL‐18 can induce high concentrations of IFN‐γ, causing liver damage.

In the existing medical reports, the main risk factors for liver cancer include infection with HBV and HCV, exposure to substances that are toxic to the liver, drinking and smoking, and chronic inflammation of the liver mediated by the immune system. In addition, a variety of inflammatory cytokines, such as IL‐1β, IL‐18, IL‐6,^23^ and IL‐17,^24^ are involved in chronic liver inflammation and can cause tumors. From the clinical parameters of patients provided by the hospital, it was observed that the vast majority of liver cancer patients also suffer from hepatitis B. In this study, the IL‐18 concentration (1048.88; 730.04–1185.10 pg/mL) in the serum of patients with liver cancer was higher than that of healthy controls and patients with four other liver diseases, indicating that the IL‐18 concentration can reflect liver damage severity to a certain extent. In the serum of liver cancer patients, IL‐18 was significantly positively correlated with ALT, GT, LD, PA, and AFU (*p* < 0.05), indicating that the increase of IL‐18 was related to liver injury. It was highly significantly positively correlated with P Ⅲ NP (*p* < 0.01), which is a peptide chain of type Ⅲ procollagen molecule extending at the amino terminus. It is cleaved from the amino terminus by endonucleases when procollagen is secreted into the extracellular space and enters the blood circulation.^25^ Serum P Ⅲ NP levels increase with the synthesis of collagen fibers in the liver, and these levels were found to be significantly increased in patients with liver cancer, suggesting that IL‐18 may be involved in the process of liver fibrosis. However, as a classic marker for the diagnosis of liver cancer‐AFP,^26^ its correlation with IL‐18 is not significant. AFP inhibits the maturation of dendritic cells (DC) and induces their apoptosis, so that antigens cannot be presented to T cells by DC cells, thereby freeing cancer cells from immune surveillance, indicating that IL‐18 does not participate in this cellular immune process.

The concentration of IL‐18 in the serum of fatty liver patients (911.97; 775.55–1130.03 pg/mL) is higher than that of healthy controls (483.09; 402.52–599.70 pg/mL), and its pathogenesis is more complicated. It has been clearly reported that excessive accumulation of fat will increase the concentration of IL‐18 and promote liver cell damage associated with fatty liver.^26^, ^27^ IFN‐γ produced by IL‐18 has the effect of strongly inducing the development of fatty liver in rats.^28^ In the process of hepatic steatosis, the production of proinflammatory mediators such as TNF‐α and IL‐6 contributes to the activation Kupffer cells.^29^ The activation of these cells helps to produce large amounts of IL‐18. In the serum of fatty liver patients, the GT concentration was significantly increased, suggesting that the enzyme is hypersynthesized in the liver and participates in liver damage together with IL‐18. There was no correlation between IL‐18 levels and LDL, TRIG, and TC, but was negatively correlated with HDL (*r* = −0.697, *p* < 0.01). The decrease of HDL concentration leads to a decline in cholesterol transport from the peripheral tissues back to the liver for processing. Decomposition increases the accumulation of harmful substances such as LDL, cholesterol, and TRIG in the liver, and the liver is more likely to develop fatty liver disease.

Importantly, we observed a significant positive correlation between TIM‐3 and IL‐18 concentration in the serum of patients with liver disease (*r* = 0.275, *p* < 0.01). Galectin‐9(Gal‐9), the ligand of TIM‐3, is widely distributed in various tissues, particularly the liver.^30^ The combination of Gal‐9 and TIM‐3 can induce Th1 cell apoptosis, while the IL‐18/IL‐12 signaling pathway promotes the Th1 immune response. Further, increased IL‐18 serum concentration promotes the Th1 immune response, which, in turn, releases IFN‐γ, thus promoting the inflammatory response. The presence of IFN‐γ and subsequently the inflammatory response lead to an increase in TIM‐3/Gal‐9, which act as a Th1 response inhibitor.

Changes such as hepatocyte swelling and necrosis caused by liver diseases will increase the permeability of the liver, capillaries, and blood vessels, making it easy for indexes such as DBIL, AST, and ALT to flow back into the blood from the liver cells and bile ducts.^31^ In patients with nonalcoholic fatty liver, the detection indexes of ALT, AST, and GT were found to be significantly higher than those of healthy controls (*p* < 0.01).^32^ In our study, the clinical indicators of ALT, LD, DBIL, TP, AST, and GT of 5 liver diseases were significantly positively correlated with IL‐18. The concentration of IL‐18 in each of the five liver diseases is different, which provides a new detection index for clinical diagnosis. The IL‐18 concentration in patients with autoimmune hepatitis did not increase significantly (*p* > 0.05). The combined detection of multiple clinical indicators suggests that a series of physiological changes will occur when the liver develops lesions, and this is worthwhile of an in‐depth analysis.

To our knowledge, this is the first study to apply the TRFIA method for the detection of IL‐18 concentration in the serum of patients with liver diseases. We found that the IL‐18 serum concentration of patients with various liver diseases differed compared to the healthy controls. Furthermore, the TRFIA method used here is more sensitive than the traditional ELISA and also exhibits good specificity. This method is relatively simple to use, efficient, and cost‐effective; thus, it might prove useful for both laboratory and clinical settings.

## 5 FUNDING SOURCES

This work was supported by Wuxi Key Laboratory of Infectious Disease Prevention and Control [CXPT(SYS)001]; the Social Development Fund of Zhejiang Province [LGF20H200008]; and the Key Research and Development Program of Zhejiang Province [2020C03066].

## CONFLICT OF INTEREST

None.

## Data Availability

All the data that related to this study are available from the corresponding author upon reasonable request.

## References

[1] Yamanaka R , Honma J , Tsuchiya N , Yajima N , Kobayashi T , Tanaka R . Tumor lysate and IL‐18 loaded dendritic cells elicits Th1 response, tumor‐specific CD8+ cytotoxic T cells in patients with malignant glioma. J Neurooncol. 2005;72:107‐113. 15925989 10.1007/s11060-004-3550-9

[2] Dinarello CA . The IL‐1 family and inflammatory diseases. Clin Exp Rheumatol. 2002;20:S1‐13. 14989423

[3] Dinarello CA . Interleukin‐18 and the pathogenesis of inflammatory diseases. Semin Nephrol. 2007;27:98‐114. 17336692 10.1016/j.semnephrol.2006.09.013

[4] Martinon F , Tschopp J . Inflammatory caspases and inflammasomes: Master switches of inflammation. Cell Death Differ. 2007;14:10‐22. 16977329 10.1038/sj.cdd.4402038

[5] Krishnan SM , Sobey CG , Latz E , Mansell A , Drummond GR . IL‐1β and IL‐18: inflammatory markers or mediators of hypertension? Br J Pharmacol. 2014;171:5589‐5602. 25117218 10.1111/bph.12876 PMC4290704

[6] Choudhary A , Basu S , Dey SK , Rout JK , Das RK , Dey RK . Association and prognostic value of serum Cystatin C, IL‐18 and Uric acid in urological patients with acute kidney injury. Clin Chim Acta. 2018;482:144‐148. 29627485 10.1016/j.cca.2018.04.005

[7] Uzan B , Poglio S , Gerby B , et al. Interleukin‐18 produced by bone marrow‐derived stromal cells supports T‐cell acute leukaemia progression. EMBO Mol Med. 2014;6:821‐834. 24778454 10.1002/emmm.201303286 PMC4203358

[8] Mallat Z , Corbaz A , Scoazec A , et al. Expression of interleukin‐18 in human atherosclerotic plaques and relation to plaque instability. Circulation. 2001;104:1598‐1603. 11581135 10.1161/hc3901.096721

[9] Bruun JM , Stallknecht B , Helge JW , Richelsen B . Interleukin‐18 in plasma and adipose tissue: effects of obesity, insulin resistance, and weight loss. Eur J Endocrinol. 2007;157:465‐471. 17893261 10.1530/EJE-07-0206

[10] Hivert MF , Sun Q , Shrader P , Mantzoros CS , Meigs JB , Hu FB . Circulating IL‐18 and the risk of type 2 diabetes in women. Diabetologia. 2009;52:2101‐2108. 19669125 10.1007/s00125-009-1455-z PMC3758765

[11] Zhu M , Rong X , Li M , Wang S . IL‐18 and IL‐35 in the serum of patients with sepsis thrombocytopenia and the clinical significance. Exp Ther Med. 2020;19:1251‐1258. 32010296 10.3892/etm.2019.8347 PMC6966114

[12] Ramezani A , Hasanjani Roshan MR , Kalantar E , et al. Association of human leukocyte antigen polymorphism with outcomes of hepatitis B virus infection. J Gastroenterol Hepatol. 2008;23:1716‐1721. 18761557 10.1111/j.1440-1746.2008.05482.x

[13] Bao J , Lu Y , Deng Y , et al. Association between IL‐18 polymorphisms, serum levels, and HBV‐related hepatocellular carcinoma in a Chinese population: a retrospective case–control study. Cancer Cell Int. 2015;15:72. 26213495 10.1186/s12935-015-0223-z PMC4513629

[14] Wang H , Zhai X , Liu T , et al. Development of a novel immunoassay for the simple and fast quantitation of neutrophil gelatinase‐associated lipocalin using europium(iii) chelate microparticles and magnetic beads. J Immunol Methods. 2019;470:15‐19. 31004578 10.1016/j.jim.2019.04.004

[15] Huang B , Zhang Y , Wang L , et al. Phospholipase A2 receptor antibody IgG4 subclass improves sensitivity and specificity in the diagnosis of idiopathic membranous nephropathy. Kidney Blood Press Res. 2019;44:848‐857. 31242492 10.1159/000500456

[16] Chen M , Wang L , Wang Y , et al. Soluble Tim3 detection by time‐resolved fluorescence immunoassay and its application in membranous nephropathy. J Clin Lab Anal. 2020;34:e23248. 32077157 10.1002/jcla.23248 PMC7307342

[17] Yang SQ , Lin HZ , Lane MD , Clemens M , Diehl AM . Obesity increases sensitivity to endotoxin liver injury: Implications for the pathogenesis of steatohepatitis. Proc Natl Acad Sci. 1997;94:2557‐2562. 9122234 10.1073/pnas.94.6.2557 PMC20127

[18] Diehl AM . Cytokine regulation of liver injury and repair. Immunol Rev. 2000;174:160‐171. 10807515 10.1034/j.1600-0528.2002.017411.x

[19] Jiang H , Cao F , Cao H , Rao Q , Yang Y . Associations of human leukocyte antigen and interleukin‐18 gene polymorphisms with viral load in patients with hepatitis B infection. Medicine (Baltimore). 2018;97(30):e11249. 30045250 10.1097/MD.0000000000011249 PMC6078658

[20] Zhang T , Kawakami K , Qureshi MH , Okamura H , Kurimoto M , Saito A . Interleukin‐12 (IL‐12) and IL‐18 synergistically induce the fungicidal activity of murine peritoneal exudate cells against Cryptococcus neoformans through production of gamma interferon by natural killer cells. Infect Immun. 1997;65:3594‐3599. 9284124 10.1128/iai.65.9.3594-3599.1997 PMC175511

[21] Kim WR , Brown RS , Terrault NA , El‐Serag H . Burden of liver disease in the United States: Summary of a workshop. Hepatology. 2002;36:227‐242. 12085369 10.1053/jhep.2002.34734

[22] Sharma A , Chakraborti A , Das A , Dhiman RK , Chawla Y . Elevation of interleukin‐18 in chronic hepatitis C: implications for hepatitis C virus pathogenesis. Immunology. 2009;128:e514‐e522. 19740312 10.1111/j.1365-2567.2008.03021.x PMC2753952

[23] Li S , Sun R , Chen Y , Wei H , Tian Z . Tlr2 limits development of hepatocellular carcinoma by reducing il18‐mediated immunosuppression. Cancer Res. 2015;75:986. 25600646 10.1158/0008-5472.CAN-14-2371

[24] Gu FM , Li QL , Gao Q , et al. IL‐17 induces AKT‐dependent IL‐6/JAK2/STAT3 activation and tumor progression in hepatocellular carcinoma. Mol Cancer. 2011;10:150. 22171994 10.1186/1476-4598-10-150 PMC3310750

[25] Zhang XL . The clinical significance of combined detection of serum hyaluronic acid, type Ⅲ procollagen amino terminal peptide, type Ⅳ collagen and laminin in the auxiliary diagnosis of chronic hepatitis B liver fibrosis and cirrhosis. J Clin Ration Use. 2017;010(025):114‐116.[in Chinese].

[26] Park SJ , Jang JY , Jeong SW , et al. Usefulness of AFP, AFP‐L3, and PIVKA‐II, and their combinations in diagnosing hepatocellular carcinoma. Medicine (Baltimore). 2017;96(11):e5811. 28296720 10.1097/MD.0000000000005811 PMC5369875

[27] Sun J , Xu Y , Dai Z , Sun Y . Intermittent high glucose stimulate MCP‐l, IL‐18, and PAI‐1, but inhibit adiponectin expression and secretion in adipocytes dependent of ROS. Cell Biochem Biophys. 2009;55:173‐180. 19756411 10.1007/s12013-009-9066-3

[28] Dinarello C , Kaplanski G . Indeed, IL‐18 is more than an inducer of IFN‐γ. J Leukoc Biol. 2018;104(2):237‐238. 29733453 10.1002/JLB.CE0118-025RR PMC6290467

[29] Marchisello S , Di Pino A , Scicali R , et al. Pathophysiological, molecular and therapeutic issues of nonalcoholic fatty liver disease: an overview. Int J Mol Sci. 2019;20(8):1948. 10.3390/ijms20081948 PMC6514656 31010049

[30] Wada J , Kanwar YS . Identification and characterization of Galectin‐9, a novel β‐Galactoside‐binding mammalian lectin. J Biol Chem. 1997;272:6078‐6086. 9038233 10.1074/jbc.272.9.6078

[31] Wang KL , Zhong J . The clinical significance of serum bilirubin components in the diagnosis of liver diseases. Chinese Laboratory Diagnostics 1997;2:21‐23.[in Chinese]

[32] Sogabe M , Okahisa T , Kurihara T , et al. Differences among patients with and without nonalcoholic fatty liver disease having elevated alanine aminotransferase levels at various stages of metabolic syndrome. PLoS One. 2020;15(8):e0238388. 32866186 10.1371/journal.pone.0238388 PMC7458345

